# Coupling of lysosomal and mitochondrial membrane permeabilization in trypanolysis by APOL1

**DOI:** 10.1038/ncomms9078

**Published:** 2015-08-26

**Authors:** Gilles Vanwalleghem, Frédéric Fontaine, Laurence Lecordier, Patricia Tebabi, Kristoffer Klewe, Derek P. Nolan, Yoshiki Yamaryo-Botté, Cyrille Botté, Anneke Kremer, Gabriela Schumann Burkard, Joachim Rassow, Isabel Roditi, David Pérez-Morga, Etienne Pays

**Affiliations:** 1 Laboratory of Molecular Parasitology, IBMM, Université Libre de Bruxelles (ULB), 12 rue des Prof Jeener et Brachet, B-6041 Gosselies, Belgium; 2 Institute of Biochemistry and Pathobiochemistry, Ruhr-Universität Bochum, Universitätsstrasse 150, 44780 Bochum, Germany; 3 Molecular Parasitology Group, School of Biochemistry and Immunology, Trinity College Dublin, Dublin 2, Ireland; 4 Apicolipid Group, CNRS, Laboratoire Adaptation et Pathogénie des Microorganismes UMR5163/ Institut Albert Bonniot CRI Inserm/UJF U823, CNRS, Institut Jean Roget, 38700 La Tronche, France; 5 IRC/VIB Bio Imaging Core, Gent, Technologiepark 927, B-9052 Gent, Belgium; 6 Institute of Cell Biology, University of Bern, Baltzerstrasse 4, CH-3012 Bern, Switzerland; 7 Center for Microscopy and Molecular Imaging (CMMI), Université Libre de Bruxelles (ULB), 8 rue Adrienne Bolland, B-6041 Gosselies, Belgium; 8 Walloon Excellence in Life sciences and Biotechnology (WELBIO), Wavre, Belgium

## Abstract

Humans resist infection by the African parasite *Trypanosoma brucei* owing to the trypanolytic activity of the serum apolipoprotein L1 (APOL1). Following uptake by endocytosis in the parasite, APOL1 forms pores in endolysosomal membranes and triggers lysosome swelling. Here we show that APOL1 induces both lysosomal and mitochondrial membrane permeabilization (LMP and MMP). Trypanolysis coincides with MMP and consecutive release of the mitochondrial *Tb*EndoG endonuclease to the nucleus. APOL1 is associated with the kinesin *Tb*KIFC1, of which both the motor and vesicular trafficking VHS domains are required for MMP, but not for LMP. The presence of APOL1 in the mitochondrion is accompanied by mitochondrial membrane fenestration, which can be mimicked by knockdown of a mitochondrial mitofusin-like protein (*Tb*MFNL). The BH3-like peptide of APOL1 is required for LMP, MMP and trypanolysis. Thus, trypanolysis by APOL1 is linked to apoptosis-like MMP occurring together with *Tb*KIFC1-mediated transport of APOL1 from endolysosomal membranes to the mitochondrion.

## Abstract (web summary)

The human serum protein apolipoprotein L1 (APOL1) is taken up by trypanosomes where it triggers cell death, forming pores in endolysosomal membranes. Vanwalleghem *et al.* show that APOL1 triggers both lysosomal and mitochondrial membrane permeabilization, and that the latter is responsible for trypanolysis.

## Graphical abstract

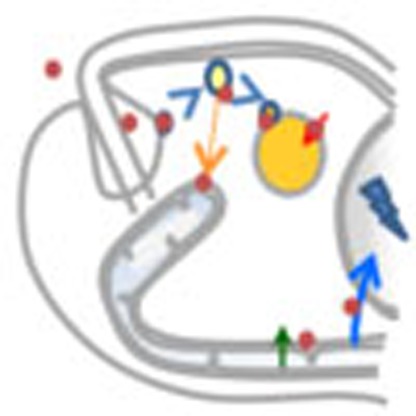

The protozoan flagellate *Trypanosoma brucei brucei* is the prototype of African trypanosomes, parasites able to infect a wide variety of mammalian hosts. In humans, *T. b. brucei* cannot develop infection, but the two subspecies *Trypanosoma brucei rhodesiense* and *Trypanosoma brucei gambiense* cause a lethal disease termed sleeping sickness. This differential infection ability results from the presence in normal human serum (NHS) of the trypanolytic factor apolipoprotein L1 (APOL1), which kills *T. b. brucei* but not *T. b. rhodesiense* and *T. b. gambiense*^1^,^2^,^3^. APOL1 is the only extracellular member of a family of six proteins that appear to play a role in the control of cell death on infection by pathogens^2^. APOL1 contains an N-terminal ionic pore-forming domain contiguous to a membrane-addressing motif, together with a long amphipathic α-helix in the C-terminal region^1^,^3^. This protein exhibits characteristics of pro-apoptotic BCL2 proteins, such as a BCL2 homology 3 (BH3)-like peptide and BAX-like ion channel properties^2^,^3^,^4^. In trypanosomes APOL1 is taken up through endocytosis that involves the haptoglobin–haemoglobin surface receptor TbHpHbR (refs 5, 6). On acidification in the endocytic pathway APOL1 is inserted in vacuolar membranes where its pore-forming domain triggers an influx of chloride ions resulting in osmotic swelling of the lysosome^3^,^6^. *T. b. rhodesiense* and *T. b. gambiense* inhibit APOL1 activity by expressing specific resistance proteins that, respectively, neutralize the toxin by direct interaction with the C-terminal helix or protect endosomal membranes against APOL1 insertion^1^,^6^,^7^,^8^.

Apart from the evidence of lysosome swelling, the mechanism underlying parasite lysis by APOL1 was not clear. In this work we attempted to evaluate the nature of the lysosome membrane pores formed by APOL1, and their involvement in trypanosome lysis. Surprisingly, we discovered that APOL1 also generates mitochondrial membrane pores, and that a particular kinesin is required for this process. Trypanosome death was found to coincide with the APOL1-triggered release of a mitochondrial endonuclease and subsequent DNA fragmentation.

## Results

### Involvement of lysosome swelling in lysis

**Figure 1:**
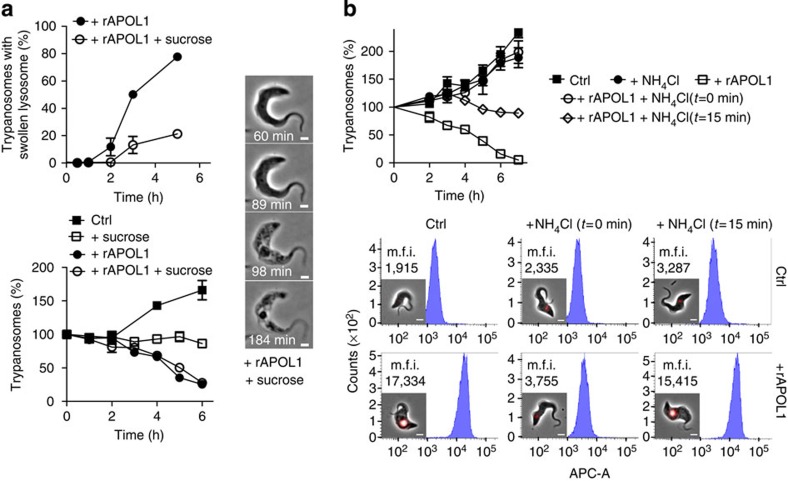
Lysosomal swelling and trypanolysis in control and 10 μg ml^−1^ rAPOL1-treated *T. brucei*. (**a**) Effect of 0.175 M sucrose (error bars: s.e.m.; three replicates; *n*=3; scale bar, 2 μm). (**b**) Effect of 20 mM NH_4_Cl. The FACS panels illustrate the fluorescence intensity of trypanosomes loaded with Texas Red-labelled 10 kDa dextran beads, as a way to monitor lysosome swelling by fluorescence measurement (error bars: s.e.m.; three replicates; *n*=3; m.f.i., mean fluorescent intensity of 10,000 gated cells; scale bar, 2 μm).

We evaluated whether trypanolysis by recombinant APOL1 (rAPOL1) could result from lysosomal swelling. Increasing the osmotic strength of the medium by addition of sucrose largely prevented lysosomal swelling without affecting trypanolysis (Fig. 1a). As expected, the presence of NH_4_Cl together with rAPOL1 inhibited lysosomal swelling and trypanolysis due to elevation of endosomal pH^3^,^9^ (Fig. 1b). However, addition of NH_4_Cl after 15-min incubation with rAPOL1 resulted in resistance to trypanolysis despite lysosome swelling (Fig. 1b). Therefore, lysosomal swelling was not responsible for trypanolysis.

### Nature of the lysosomal pores

**Figure 2:**
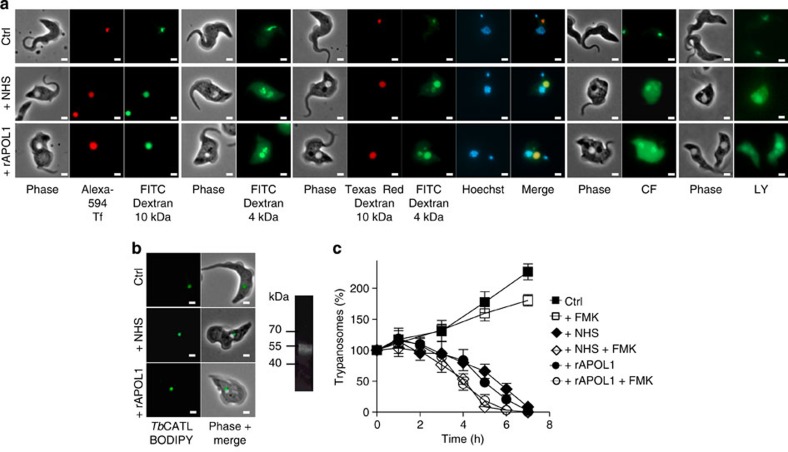
LMP and trypanolysis in control and 30% NHS- or 10 μg ml^−1^ rAPOL1-treated *T. brucei*. (**a**) Intracellular localization of fluorescent markers. Tf, transferrin; CF, carboxyfluorescein; LY, Lucifer yellow (1 h incubation; scale bar, 2 μm; same exposure time in all panels). (**b**) Intracellular localization of *Tb*CATL–BODIPY (1 h incubation; scale bar, 2 μm). Right panel: *Tb*CATL–BODIPY fluorescence in a trypanosome extract submitted to SDS–polyacrylamide gel electrophoresis. (**c**) Trypanolysis following 30-min preincubation with 10 mM cathepsin inhibitor FMK-024 (error bars: s.e.m.; three replicates; *n*=3).

To characterize the lysosomal pores formed by APOL1, we incubated *T. brucei* with different fluorescent markers. In the absence of NHS or rAPOL1 the probes remained within the lysosome, but with NHS or rAPOL1 all markers except the 10-kDa dextran spread into the cytoplasm and nucleus (Fig. 2a; Supplementary Fig. 1). Thus, APOL1 appeared to permeabilize the lysosomal membrane for molecules <10 kDa. Apoptotic lysosomal membrane permeabilization (LMP) is thought to allow cathepsin release^10^, so we incubated trypanosomes with fluorescent *Tb*CATL cathepsin^11^. *Tb*CATL–BODIPY accumulated intact within the lysosome, with no evidence for leakage on incubation with NHS or rAPOL1 despite lysosomal swelling (Fig. 2b). Moreover, incubation with the cathepsin inhibitor FMK-024 did not prevent APOL1-mediated trypanolysis but rather accelerated this process (Fig. 2c), indicating, like other studies^8^,^11^,^12^, that trypanolysis cannot result from cathepsin release and/or activity.

### APOL1-induced effects in the mitochondrion

**Figure 3:**
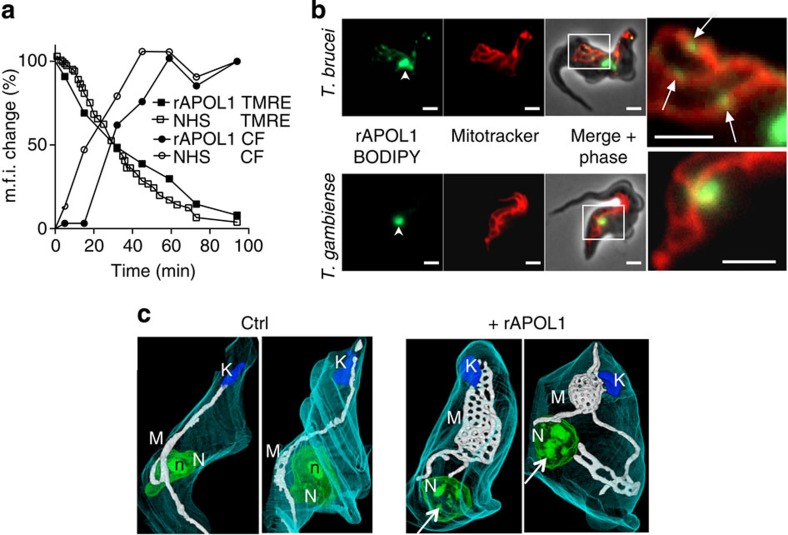
Mitochondrial membrane effects in control and 30% NHS- or 10 μg ml^−1^ rAPOL1-treated *T. brucei*. (**a**) Kinetics of tetramethylrhodamine ethyl ester perchlorate (TMRE) and carboxyfluorescein (CF) fluorescence changes (% maximum) measured by FACS on 10,000 trypanosomes as respective indicators of mitochondrial membrane depolarization and lysosome swelling (100 and 0% m.f.i. values=8,237 and 289 for TMRE, 2,734 and 200 for CF). (**b**) Intracellular localization of rAPOL1–BODIPY (1 h incubation; arrowhead and arrows: lysosomal and mitochondrial APOL1; bars=2 μm). (**c**) Focused ion beam–scanning electron microscope tomography (1 h incubation with rAPOL1). K, kinetoplast; M, mitochondrion; N, nucleus; n, nucleolus; arrows, heterochromatin patches. Videos are available in Supplementary Materials.

**Figure 4:**
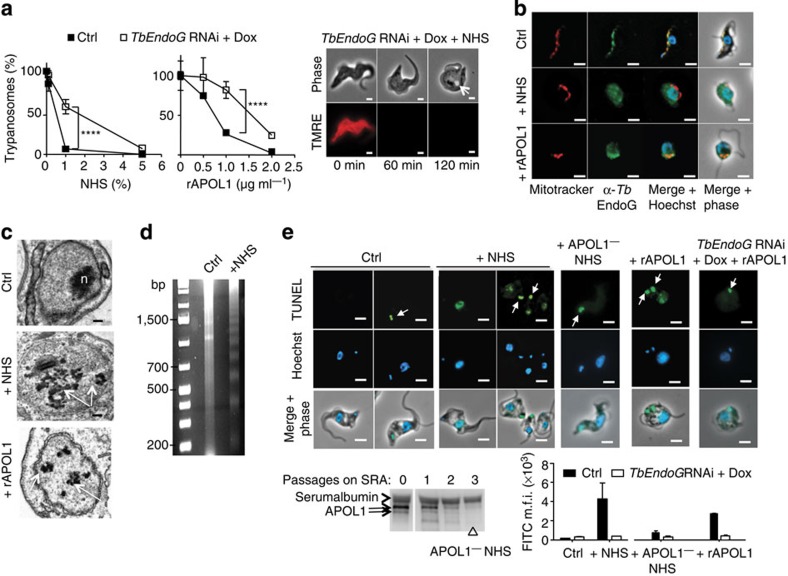
MMP and trypanolysis in control and NHS- or rAPOL1-treated *T. brucei.* (**a**) Effect of *TbEndoG* RNAi (2 days with Dox) (overnight incubation with NHS or rAPOL1; error bars: s.e.m.; three replicates; *n*=3; statistical significance: two-way analysis of variance, Sidak *post hoc*). Right panel: lysosomal swelling and tetramethylrhodamine ethyl ester perchlorate staining in *TbEndoG* RNAi cells treated with 30% NHS (scale bars, 2 μm; arrow: swollen lysosome). (**b**) Detection of *Tb*EndoG in *T. brucei* following 1-h incubation with 30% NHS or 10 μg ml^−1^ rAPOL1 (Hoechst labels DNA; scale bars, 2 μm); (**c**–**e**) apoptotic-like features in the nucleus following 1-h incubation with 30% NHS or 10 μg ml^−1^ rAPOL1: (**c**) transmission electron microscopy (scale bars, 200 nm; n, nucleolus; arrows: heterochromatin patches); (**d**) electrophoretic migration of purified genomic DNA (ethidium bromide staining); (**e**) terminal deoxynucleotidyl transferase-mediated dUTP nick-end labelling of DNA (TUNEL) in control (Ctrl) or *TbEndoG* RNAi cells; the FITC signal of fluorescent nucleotides transferred at cleaved DNA ends is directly proportional to DNA degradation (arrows: kinetoplast DNA labelling in dividing cells; scale bars, 2 μm; error bars: s.e.m.; three replicates; *n*=3; statistical significance: two-way analysis of variance, Sidak *post hoc*). This experiment includes 30% NHS depleted of APOL1 following successive elution on SRA beads (APOL1^−^ NHS), as illustrated in a western blot incubated with anti-APOL1 antibodies.

LMP was accompanied by depolarization of the mitochondrial membrane (Fig. 3a), which occurred together with the transfer of some rAPOL1 to the mitochondrion (Fig. 3b; Supplementary Fig. 2). This transfer required the insertion of APOL1 into endolysosomal membranes as it was not observed in *T. b. gambiense* where APOL1 cannot be inserted into these membranes^6^,^8^ (Fig. 3b; Supplementary Fig. 2). The presence of APOL1 was also accompanied by mitochondrial membrane fenestration evoking a fission defect (Fig. 3c). In other unicellular eukaryotes cell death can result from mitochondrial membrane permeabilization (MMP) triggering the release of the mitochondrial endonuclease G (EndoG) to the nucleus^13^. Knockdown of *Tb*EndoG expression following induction of *TbEndoG* RNAi^14^ (Supplementary Fig. 3a) led to resistance to trypanolysis by NHS or rAPOL1 even though lysosomal swelling and mitochondrial depolarization occurred (Fig. 4a). Moreover, NHS or rAPOL1 treatment induced *Tb*EndoG release from the mitochondrion (Fig. 4b; Supplementary Fig. 3b) along with evidence of nuclear alterations. These included chromatin condensation into heterochromatin patches (Figs 3c and 4c), DNA laddering in fragments resulting from inter-nucleosomal cleavages (Fig. 4d) and strong increase of DNA end labelling in the TUNEL assay, reflecting DNA fragmentation (Fig. 4e). DNA fragmentation was strictly linked to the presence of APOL1, since it did not occur in NHS depleted of APOL1 (Fig. 4e). *Tb*EndoG was involved in this fragmentation, since knockdown of *Tb*EndoG expression abolished NHS- or rAPOL1-linked DNA end labelling (Fig. 4e). Thus, APOL1-mediated trypanolysis clearly coincided with MMP.

### Identification of a kinesin involved in LMP-MMP coupling

**Figure 5:**
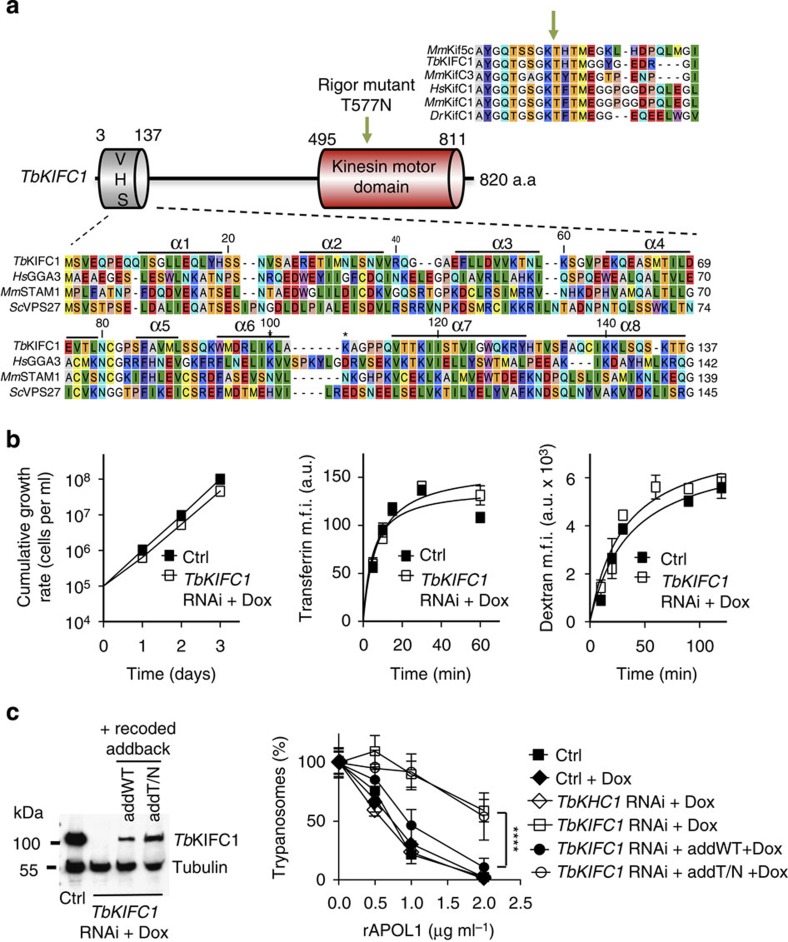
Involvement of *Tb*KIFC1 in trypanolysis by APOL1. (**a**) Scheme illustrating the presence of VHS and motor domains in *Tb*KIFC1, with indication of the residues mutated in the addback versions of the gene: T577N (arrow)^17^ and K94/97A (asterisks)^19^. The sequence alignments show similarities between the motor (above) and VHS (below) domains of *Tb*KIFC1 and the corresponding domains from various proteins (UniProt nomenclature: *Hs*GGA3=Q9NZ52; *Mm*STAM1=P70297; *Sc*VPS27=P40343; *Tb*KIFC1=Q388B7; *Mm*Kif5C=P28738; *Mm*KifC3=O35231; *Hs*KifC1=Q9BW19; *Mm*KifC1=Q9QWT9; *Dr*KifC1=Q1LUU7). The eight VHS helices are indicated above the sequences. (**b**) Trypanosome growth rate and fluorescent marker uptake on *TbKIFC1* RNAi (1 day with Dox) (error bars: s.e.m.; three replicates; *n*=3). (**c**) *Tb*KIFC1 involvement in trypanolysis (left: western blotting; right: trypanolysis after overnight incubation with rAPOL1) (*Tb*KHC1, control unrelated kinesin, *T. brucei* kinesin heavy chain^40^; addT/N, addback T577N; error bars: s.e.m.; three replicates; *n*=3; statistical significance: two-way analysis of variance, Sidak *post hoc*).

**Figure 6:**
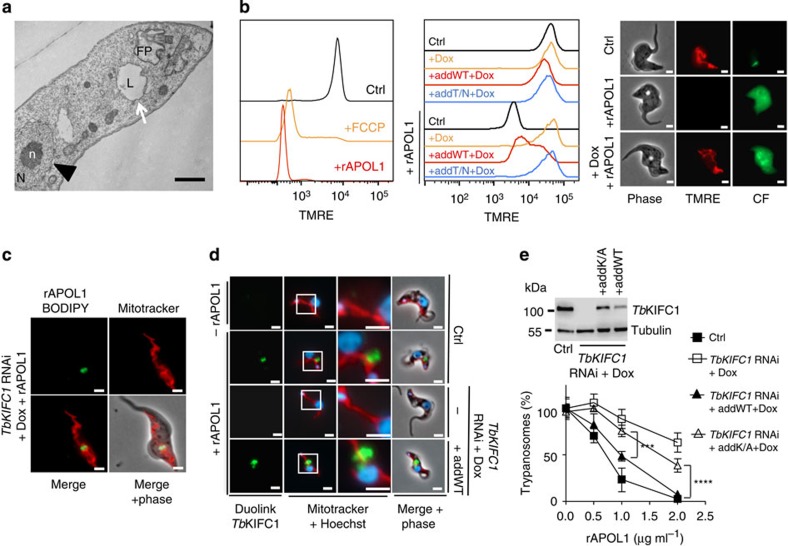
*TbKIFC1* RNAi-mediated uncoupling between LMP and MMP following 1-h incubation with 10 μg ml^−1^ rAPOL1. (**a**) Transmission electron microscopy of doxyxycline-induced *TbKIFC1* RNAi cells (FP, flagellar pocket; L, lysosome; N, nucleus; n, nucleolus; arrowhead and arrow: normal nucleus and swollen lysosome, respectively; scale bar, 1 μm); (**b**) Tetramethylrhodamine ethyl ester perchlorate (TMRE) and carboxyfluorescein (CF) staining of control (Ctrl), *TbKIFC1* RNAi and addback cell lines (FACS of 40,000 trypanosomes; left: effect of 10 μM proton ionophore carbonyl cyanide-4-(trifluoromethoxy) phenylhydrazone (FCCP) (15 min incubation); scale bars, 2 μm). (**c**) Intracellular localization of rAPOL1–BODIPY (10 μg ml^−1^) in *TbKIFC1* RNAi cells (scale bars, 2 μm). (**d**) Duolink labelling using the anti-*Tb*KIFC1 and anti-APOL1 antibodies (scale bars, 2 μm). (**e**) *Tb*KIFC1 involvement in trypanolysis (top: western blotting; bottom: trypanolysis after overnight incubation with rAPOL1) (addK/A, addback K94A/K97A; error bars: s.e.m.; three replicates; *n*=3; statistical significance: two-way analysis of variance, Sidak *post hoc*).

To identify trypanosome components involved in APOL1-induced MMP, we screened a trypanosome RNAi library for resistance to NHS^15^. The gene encoding the C-terminal kinesin *Tb*KIFC1 (ref. 16), which contains an N-terminal VPS-27, Hrs and STAM (VHS) domain typically involved in membrane recognition for vesicular trafficking (Fig. 5a), was selected four times from 12 independent experiments. *Tb*KIFC1 depletion by RNAi (Supplementary Fig. 4a) did not affect the *in vitro* growth rate, receptor-mediated endocytosis, fluid phase uptake (Fig. 5b) or endolysosomal pH (Supplementary Table 1), but conferred important resistance to rAPOL1 (Fig. 5c). To verify the specificity of the RNAi phenotype, we transfected a recoded version of the *TbKIFC1* gene in the RNAi cell line, allowing this gene to escape RNAi targeting and to resume expression of *Tb*KIFC1. This experiment restored sensitivity to rAPOL1 unless the ATP-binding motif of the kinesin was mutated^17^ (Fig. 5c; Supplementary Fig. 4a), indicating that the motor domain of *Tb*KIFC1 is involved in cellular sensitivity to APOL1. Significantly, when *Tb*KIFC1 was knocked down, rAPOL1 no longer affected nuclear structure or mitochondrial membrane potential despite lysosomal swelling and LMP (Fig. 6a,b; Supplementary Fig. 5a,b). Moreover, in these cells rAPOL1 was only observed in the lysosome and no longer in the mitochondrion (Fig. 6c; Supplementary Fig. 5c). These results demonstrated that *Tb*KIFC1 motor activity is crucially involved in coupling LMP to MMP. *Tb*KIFC1 delocalization occurring on NH_4_Cl treatment^16^ could explain the uncoupling between LMP and trypanolysis observed when NH_4_Cl is added after rAPOL1 uptake (Fig. 1b). Consistent with these views, the Duolink ligation assay^18^ revealed a close proximity (below 40 nm) between some rAPOL1 and *Tb*KIFC1 (Fig. 6d; Supplementary Fig. 6). *Tb*KIFC1 did not localize in the endocytic compartment (Supplementary Fig. 4b), but trypanolysis was affected following mutations of *Tb*KIFC1 VHS residues (K94A/K97A) predicted to interact with endosomes^19^ (Fig. 6e). Thus, *Tb*KIFC1 could transport endolysosomal membrane-inserted APOL1 to the mitochondrion.

### Uncoupling trypanolysis from mitochondrial fenestration

**Figure 7:**
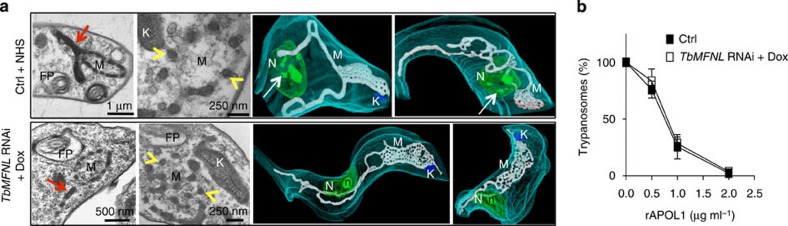
Mitochondrial targeting by APOL1. (**a**) Mitochondrion remodelling in 30% NHS-treated cells or *TbMFNL* RNAi cells (2 days+Dox). Left panels: transmission electron microscopy (red arrows and yellow arrowheads show longitudinal and cross-sections of fenestrated mitochondria). Right panels: focused ion beam–scanning electron microscope tomography. FP, flagellar pocket; K, kinetoplast; M, mitochondrion; N, nucleus; n, nucleolus; arrows, heterochromatin patches. Videos are available in Supplementary Materials. (**b**) Effect of *TbMFNL* RNAi (2 days with Dox) on trypanolysis after overnight incubation with rAPOL1 (error bars: s.e.m.; three replicates; *n*=3).

To evaluate the possible involvement of mitochondrial fenestration in the process of trypanolysis, we attempted to identify trypanosome enzymes that could be responsible for mitochondrial fission. RNAi-mediated knockdown of a trypanosomal mitofusin-like protein that we termed *Tb*MFNL (Tb927.7.2410; Supplementary Fig. 7) mimicked the mitochondrial membrane fenestration observed with APOL1 (Fig. 7a). However, mitochondrial membrane fenestration *per se,* such as that resulting from depletion of *Tb*MFNL, was not trypanolytic. This fenestration did not cause nuclear heterochromatinization and trypanolysis, nor did it affect trypanolysis by rAPOL1 (Fig. 7a,b).

### Role of the BH3-like peptide

**Figure 8:**
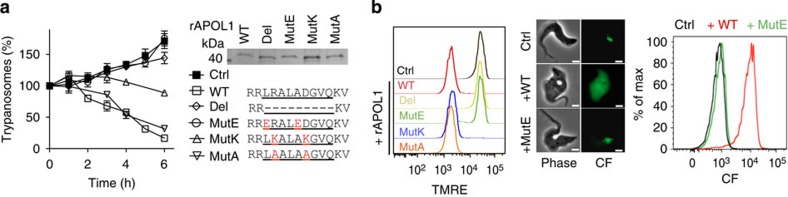
Effect of BH3 mutations on trypanolysis, LMP and MMP. (**a**) Trypanolysis by 10 μg ml^−1^ rAPOL1 mutants (right: SDS–polyacrylamide gel electrophoresis and BH3 sequence) (error bars: s.e.m.; three replicates; *n*=3). (**b**) Tetramethylrhodamine ethyl ester perchlorate and carboxyfluoresceinfluorescence on incubation with 10 μg ml^−1^ rAPOL1 (wild-type (WT) or BH3 mutants).

We generated a series of rAPOL1variants mutated in or deleted of the BH3-like peptide, including a mutation known to inactivate BH3 in mice (MutE)^20^ (Fig. 8a). All rAPOL1 effects were abrogated by deletion or MutE mutation, indicating that this peptide is required for MMP (Fig. 8a,b). With a given BH3 mutant (MutK), mitochondrial membrane depolarization occurred together with resistance to trypanolysis, suggesting membrane insertion without proper MMP (Fig. 8a,b).

**Figure 9:**
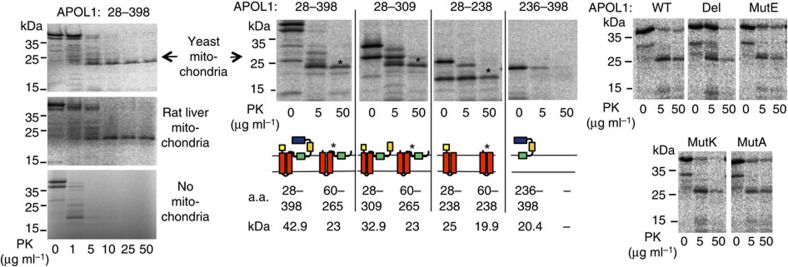
APOL1 insertion into the mitochondrial membrane of representative lower and higher eukaryotes. Radiolabelled APOL1 fragments (amino-acid numbering given above the panels) or full-size APOL1 mutants were incubated *in vitro* with isolated mitochondria, then incubated with proteinase K (PK). The polypeptide resistance to PK was evaluated by autoradiography of western blots of incubation extracts (asterisks: APOL1 fragments protected from PK following insertion into the membrane). In the central panels the domain structure of APOL1 (refs 3, 6) is schematically represented (yellow: N-terminal peptide; red: pore-forming domain; green: membrane-addressing domain; orange: hinge; blue: C-terminal helix); for each panel, the structure of the probed APOL1 polypeptide is shown at the left, and that of the PK-resistant fragment (asterisk) is shown at the right, with indication of the respective polypeptide molecular weights.

To approach the mechanism of APOL1 insertion into membranes and the possible role of the BH3-like peptide in this process, we incubated isolated mitochondria with rAPOL1 or rAPOL1 fragments. As shown in Fig. 9, under these *in vitro* conditions the rAPOL1 pore-forming domain was able to insert into the membrane of isolated mitochondria independently of the BH3-like peptide. However, these results do not allow any conclusion regarding the structure of the APOL1 pores, which may be influenced by the BH3-like peptide.

## Discussion

Our findings reveal that in *T. brucei*, APOL1 triggers apoptosis-like cell death. So far, programmed trypanosome death has been reported following oxidative stress *in vitro*^14^,^21^, but *T. brucei* trypanolysis by APOL1 does not seem to involve oxidative stress^12^ (Supplementary Fig. 8a). APOL1 uptake resulted in both LMP and MMP. A similar LMP/MMP coupling was observed in cells targeted by the pore-forming enterohaemorrhagic *Escherichia coli* hemolysin^22^ or VacA *Helicobacter pylori* toxin^23^. In higher eukaryotic cells LMP/MMP coupling is supposed to involve cathepsin release from the lysosome^10^. In trypanosomes, the lysosomal pores formed by APOL1 are too small for cathepsin release, and cathepsins do not promote trypanolysis^8^,^11^,^12^. Similarly, caspases cannot be involved because trypanosomatids lack caspases^24^. Kinetic data suggested a quick dispatching of APOL1 to both mitochondrion and lysosome. APOL1 is probably trafficked within membranes, since rAPOL1 did not reach the mitochondrion in *T. b. gambiense*, where APOL1 is prevented from endolysosomal membrane insertion^6^,^8^. As trypanolysis is clearly dependent on endocytosis^1^,^6^,^7^, the *Tb*KIFC1-mediated APOL1 targeting to the mitochondrion must follow a first uptake into endosomes. Accordingly (i) we never observed MMP without LMP; (ii) the LMP/MMP uncoupling effect of NH_4_Cl could be explained by the association of *Tb*KIFC1 with acidic vesicles^16^; (iii) in mice, KIFC1 is involved in early endosome trafficking^25^; and (iv) *Tb*KIFC1 also appears to be involved in suramin trafficking after endocytosis^26^. APOL1 did not seem to be trafficked to the plasma membrane, since rAPOL1–BODIPY was never detected in this compartment, and no evidence for plasma membrane pores could be obtained until cell death, as monitored by propidium iodide staining (Supplementary Fig. 8b). APOL1 was not trafficked through intermembrane lipid transport pathways, since knockdown of components supposedly involved in intracellular lipid trafficking had no major impact on trypanolysis (Supplementary Table 2; Supplementary Fig. 9), and *Tb*KIFC1 knockdown did not significantly affect either cellular lipid composition or sphingolipid and cholesterol turnover (Supplementary Fig. 10).

APOL1 targeting to the mitochondrion resulted in membrane fenestration mimicked by *Tb*MFNL depletion, evoking inhibition of mitochondrial fission similar to that observed on inhibition of the mitofusin-related protein DNM1 of yeast^27^ (phylogenetic relationship between *Tb*MFNL and *Sc*DNM1 is shown in Supplementary Fig. 7a). This phenotype suggests direct or indirect inhibition of the fission activity of *Tb*MFNL by APOL1, evoking the interaction occurring between the pro-apoptotic BAK and mitofusins^28^ or the mitochondrial fragmentation resulting from BAX expression in *T. brucei*^29^. Moreover, like cell death induced by apoptotic BCL2 family members trypanolysis was linked to MMP involving the BH3-like peptide of APOL1. In *T. brucei* bloodstream forms MMP could not release cytochrome c, which is absent at this stage, but triggered *Tb*EndoG release that appeared to account entirely for rAPOL1/NHS-induced nuclear DNA degradation. This finding ruled out the involvement of cytoplasmic endonucleases, like TbTatD, such as occurs during oxidative stress-induced trypanolysis^30^. However, other mitochondrial proteins could be released following MMP, and indeed the knockout of a putative trypanosomal homologue of apoptosis-inducing factor resulted in low but significant resistance to rAPOL1 (Supplementary Table 2).

**Figure 10:**
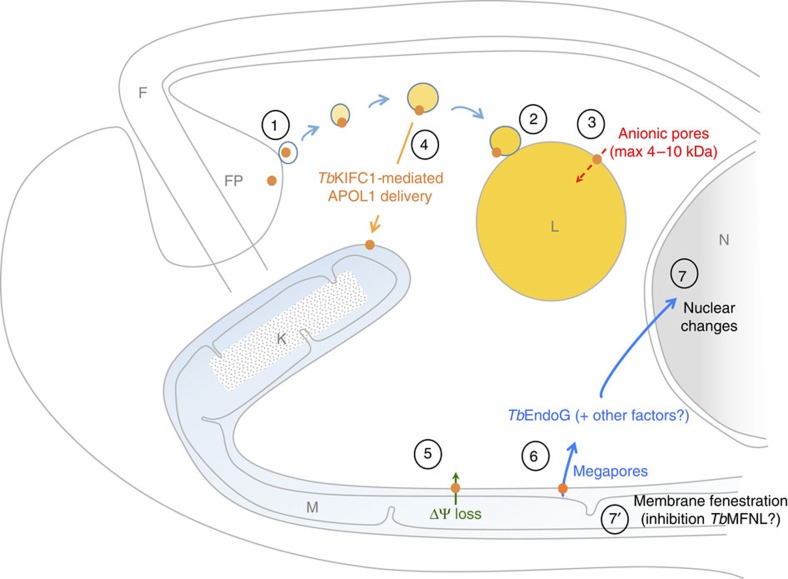
Model of APOL1-induced LMP and MMP. APOL1 is taken up by endocytosis (1) and delivered to the lysosome (2) where it causes osmotic swelling (3). *Tb*KIFC1 allows APOL1 delivery to the mitochondrion (4), leading to rapid membrane depolarization (5), *Tb*EndoG nuclease release (6) and resulting nuclear DNA fragmentation and heterochromatinization (7), concomitant with mitochondrial remodelling involving membrane fenestration (7′). The intensity of yellow staining reflects the progressive acidification in the endocytic pathway.

We conclude that trypanolysis by human serum is due to apoptosis-like MMP resulting from *Tb*KIFC1-mediated transport of APOL1 from endosomes to the mitochondrion (Fig. 10). How this transport occurs remains to be elucidated, but the insertion of APOL1 into membranes appears to be necessary, and the requirement of acidic pH for such insertion explains the necessity for APOL1 to first traffic through the endocytic system to be active^1^,^3^,^9^,^15^. Our data suggest fusion events between endosomal and mitochondrial membranes. Although such events have not been documented so far, intimate local contacts and microfusion between mitochondria and endolysosomal vacuoles have been reported^31^,^32^,^33^,^34^,^35^. In particular, in pigment cells the contacts between the lysosome-related melanosomes and mitochondria involve the mitochondrial fusion protein MFN2 (ref. 35). Given the apparent effect of APOL1 on a mitofusin-like activity, fusion of endosomal and mitochondrial membranes could be influenced by APOL1. Finally, the recent report that the conductivity of the APOL1 membrane pore is drastically enhanced when switching from low to neutral pH^36^ may provide an explanation for the differential pore activity between lysosome and mitochondrion.

## Methods

### Trypanosomes and transgenesis

The parasites were either from culture or isolated by DE-52 column purification of infected rodent blood. Stable transformations were obtained with the nucleofection method from Amaxa. Briefly, 4 × 10^7^ parasites were collected from culture of 5–8 × 10^5^ cells per ml density, centrifuged and immediately resuspended in 100 μl of Amaxa Human T cell nucleofector solution (Lonza). Transfection of 20 μg of DNA was achieved in Nucleofector Device with programme X-001. Transfected cells were resuspended in 10 ml culture medium and incubated for 16 h before addition of suitable selection drugs and dilution to 100 ml in 24-well plates. Typical experiment gave rise to 10–30 positive wells out of 96 after 6–8 days.

### *In vitro* trypanolysis and growth assays

For the overnight lysis test, parasites isolated from mice were diluted to 10^5^ cells per ml in HMI-9 medium containing 10% FCS and 10% Serum Plus, and aliquoted in triplicate in 96-well plates in the presence of various concentrations of either rAPOL1 or NHS. After 20–24-h incubation at 37 °C, parasites were counted with a haemocytometer and the ATP level was measured with CellTiter-Glo Luminescent Cell Viability Assay (Promega). For the kinetic lysis test, parasites were diluted to 5 × 10^5^ cells per ml and aliquoted in triplicate in 96-well plates in the presence of either rAPOL1 or NHS. Live cells were counted every hour with a haemocytometer. We routinely used 10 μg ml^−1^ rAPOL1 because this is the physiological concentration of APOL1 in NHS^1^. In the experiments shown in Fig. 1, 20 mM NH_4_Cl was added immediately or 15 min after initiation of lysis. *In vitro* growth assays were performed by daily dilutions of trypanosomes at 10^5^ ml^−1^ in HMI-9-supplemented medium. Normalizations were performed to reference situations.

### *T. brucei* RNAi library screening

The screening for APOL1 resistance was performed as in ref. 15, using the library described in ref. 37. The bloodstream-form RNAi library was cultivated in 10-ml flasks in HMI-9 medium containing 10% FCS and 10% Serum Plus in the presence of 1 μg ml^−1^ geneticin and 1 μg ml^−1^ hygromycin. For induction of RNAi, 1 μg ml^−1^ doxycycline was added to the culture. After 1–3 days of RNAi induction, the library was diluted in the same culture medium to 5 × 10^4^ to 5 × 10^5^ cells per ml in 10-ml flasks. After treatment with 0.01 to 3% NHS, the emergence of resistant populations was monitored. Genomic DNA was extracted (Qiamp DNA minikit, Qiagen) and RNAi inserts were amplified by PCR with Phusion DNA polymerase (Invitrogen), using the p2T7-for and p2T7-rev2 primers^37^. The PCR products were cloned into TOPO Zero blunt plasmid (Invitrogen) before bacterial transformation and sequencing. The sequences of the RNAi inserts were analysed by BLAST algorithm in NCBI, GeneDB and TriTryp databases.

The *TbKIFC1* DNA fragments selected in four independent experiments were from nucleotides 1,692–2,446, 2,438–3,130 and 1,925–2,446 (two times).

### *TbEndoG, TbMFNL* and *TbKIFC1* RNAi

A 540-bp or a 280-bp fragment derived from the *TbEndoG* or *TbMFNL* open reading frame were amplified using the primer sets: 5′-GATCCATGAGGATCCATACGTGCACTGTCATCCCA-3′ and 5′-TTCCATGACTCGAGATATAGTCACCGGGCTGCAC-3′ or 5′-GATCCATGAGGATCCTTATTTTGATGTTTGACCCC-3′ and 5′-TTCCATGACTCGAGCGGTTGAGGAACTCTTATTG-3′, respectively.

The *TbKIFC1* RNAi was built as described^16^. The amplification products were digested with *Bam*HI and *Xho*I and ligated into the p2T7-177 plasmid^38^ digested with the same enzyme mix. Linearized plasmids were transfected in single marker cell line^38^. RNAi was induced by addition of 1 μg ml^−1^ doxycycline (Duchefa).

### *TbKIFC1* recoded allele complementation

*TbKIFC1* open reading frame was recoded to avoid targeting by the *TbKIFC1* RNAi while conserving an identical coding sequence (DNA sequence identity: 67%). The recoded *TbKIFC1* was mutated at a key amino acid (T577N) to abolish its capacity to bind ATP^17^ or at K94A/K97A to tentatively abolish its capacity to bind acidic cluster-dileucine acid sorting signals^19^. Cloning into the pTSARib vector was performed as previously described^8^. *Bgl*II-restricted plasmids were transfected into the *TbKIFC1* RNAi cell line.

### Quantitative RT–PCR

Quantitative RT–PCR was performed as described^8^. Briefly, isolated RNA was treated with DNase before the reverse transcription reaction, using TURBO DNase (Ambion, Texas, USA) according to the manufacturers' instructions. The DNase was inactivated by the addition of 0.1 volume of DNase inactivation buffer for 2 min at room temperature. Complementary DNA was synthesized with Transcriptor Reverse Transcriptase (Roche Applied Science) according to the manufacturers' instructions. The following primer sets were used for mRNA quantification: 5′-GCATACGATGGCGGTTTCT-3′ and 5′-CGCTCCACAACCATTCCTATC-3′ for *TbEndoG*; 5′-AAGACACGTCGCCTCTCATT-3′ and 5′-GCACGGGTGTTGATACCTCT-3′ for *TbKIFC1*; and 5′-ATTTGGCATCCACTTTGTCA-3′ and 5′-ACCGGGTGGTAATAGAGACG-3′ for *TbMFNL.* Normalization was performed with the primer set 5′-CACCGAACTCTCCGTCAAGT-3′ and 5′-AGCCTGAATTTTCCCGTACA-3′, targeting *H2B* mRNA.

### Recombinant proteins

His6-tagged APOL1 (E28-L398) or APOL1 variants were expressed from pStaby1.2 vector (Delphi Genetics) in *E. coli* after 4 h induction at 37 °C with 1 mM isopropyl β-D-thiogalactoside. After washing, inclusion bodies were dissolved in 6 M guanidium-HCl, 50 mM phosphate buffer (pH 8.0) and incubated with Ni-NTA beads (Qiagen) for 16 h at 4 °C. All washing steps occurred at pH 8. After elution and dialysis against 20 mM acetic acid, the protein was more than 96% pure, as determined by SDS–polyacrylamide gel electrophoresis. BODIPY Fl labelling was performed by simultaneous *in vitro* transcription and translation in a TnT Coupled Wheat Germ Extract System with either FluoroTect GreenLys or Transcend tRNA (Promega).

### APOL1 depletion from NHS

The serum resistance-associated protein (SRA) of *T. b. rhodesiense* specifically and strongly interacts with APOL1, allowing specific depletion of APOL1 from NHS^1^. SRA affinity column was prepared as described in ref. 1, by incubation of 100 μg of purified recombinant SRA-His in PBS with Nickel agarose beads overnight at 4 °C. Beads were washed with PBS–1% 3-[(3-cholamidopropyl)dimethylammonio]-2-hydroxy-1-propanesulfonate (CHAPS) and equilibrated in 1 × MES buffer (0.6 M NaCl, 50 mM MES pH 5.8). A volume of 150 μl of human serum and 150 μl of 2 × MES buffer pH 5.8 were mixed and one half was incubated with the SRA-loaded beads for 2 h 30 min at 4 °C. Beads were eliminated by centrifugation and depletion was repeated for 2 h with fresh SRA beads. The depleted and undepleted sera were dialysed for 2 h at 4 °C against HMI-9 before overnight trypanolysis test. To ensure full APOL1 depletion, aliquots were analysed by western blotting before and after affinity chromatography, and additional rounds of elution were performed if necessary.

### TUNEL assay

A total of 2 × 10^7^ trypanosomes was incubated with 30% FBS or 30% NHS, then collected and fixed with 2% paraformaldehyde (PFA). The ApoAlert DNA Fragmentation Assay Kit (Clontech) was used following the manufacturer's instructions. Detection was performed either by flow cytometry using fluorescence-activated cell sorter (FACS) canto II or by immunofluorescence using a Zeiss Axioimager M2.

### Western blot analysis

Western blots were incubated for 2 h with a 1:1,000 dilution of anti-*Tb*EndoG antibody, 1:2,000 dilution of rabbit anti-APOL1 antibody (Sigma) or 1:100 dilution of mouse monoclonal anti-*Tb*KIFC1 antibody (H3) (ref. 16) in 150 mM NaCl, 0.5% (w/v) Tween 20, 20 mM Tris-HCl (pH 7.5) with 1% non-fat milk. The secondary antibodies, peroxidase-conjugated monoclonal mouse anti-rat IgGs or goat anti-rabbit IgGs (1:5,000; Serotec), were diluted in the same buffer and the bound antibodies were detected by chemiluminescence (Amersham).

### Live microscopy and flow cytometry

For the mitochondrial membrane polarity evaluation, trypanosomes were incubated for at least 15 min with 25 pM tetramethylrhodamine ethyl ester perchlorate (Life Technology) before treatment. For mitochondrial staining the mitotracker CMX ROS was added before APOL1 or NHS treatment, so that membrane depolarization occurring afterwards was without influence on the fluorescence level. For LMP and uptake measurements, trypanosomes were incubated with 0.5 mg ml^−1^ Transferrin-AF594 or -AF633, Dextran–fluorescein isothiocyanate (FITC) 10 kDa, Dextran-AF633 40 kDa (Life Technologies) or 5(6)-Carboxyfluorescein (Sigma). Lucifer yellow and Nile red (Sigma) were used at 2 mg ml^−1^ and 1.5 μg ml^−1^, respectively. For live microscopy, trypanosomes were mounted on a 1% low-melting-point agarose pad sealed with rubber glue. Cells were imaged in single plane with Axioimager M2 widefield fluorescence microscope with a × 100 Plan-APOCHROMAT 1.4 objective. For flow cytometry, cells were identified by a FSC/SSC gate and analysed with a FACS canto II. The FACS data was further analysed in FlowJo.

### Immunofluorescence

Cells were fixed and treated as described^8^. PBS-washed cells were fixed in 2% PFA for 10 min at 20 °C before being spread on poly(L-lysine)-coated slides and subsequently treated with 0.1% (v/v) Triton X-100 in Tris-buffered saline for 10 min at 20 °C. The anti-TbKIFC1 monoclonal antibody was obtained from the H3 hybridoma^16^ and used at a 1:10 dilution. The anti-*Tb*EndoG antibody was used at a 1:200 dilution. Primary antibodies were detected with an Alexa Fluor 488- or 594-conjugated goat anti-mouse or anti-rabbit IgG (Life Technologies). Cells were analysed with Zeiss Axioimager M2 widefield microscope. Deconvolution using the fast iterative algorithm (Zen Blue) was performed in Supplementary Fig. 4.

### Transmission electron microscopy

Cells were fixed for 1 h at room temperature in 2.5% glutaraldehyde in culture medium, and postfixed in 2% OsO_4_ in the same buffer. After serial dehydration in increasing ethanol concentrations, samples were embedded in agar 100 (Agar Scientific Ltd., UK) and left to polymerize for 2 days at 60 °C. Ultrathin sections (50–70-nm thick) were collected in Formvar-carbon-coated copper grids by using a Leica EM UC6 ultramicrotome and stained with uranyl acetate and lead citrate. Observations were made on a Tecnai10 electron microscope (FEI), and images were captured with an Olympus VELETA camera and processed with AnalySIS and Adobe Photoshop softwares.

### Focused ion beam–scanning electron microscope imaging

Samples were incubated in fixative (2% PFA, Applichem), 2.5% gluteraldehyde (ethylmethane sulfonate (EMS)) in 0.15 M sodium cacodylate (Sigma-Aldrich) buffer, pH7.4) at room temperature for 30 min. Fixative was removed by washing 5 × 3 min in 0.15 M cacodylate buffer and samples were incubated in 1% osmium (OsO_4_, EMS), 1.5% potassium ferrocyanide (Sigma-Aldrich) in 0.15 M cacodylate buffer for 40 min at room temperature. This was immediately followed by a second incubation in OsO4 (1% osmium in double distilled H_2_O (ddH_2_O) for 40 min at room temperature. After washing in ddH_2_O for 5 × 3 min, samples were incubated overnight at 4 °C in 1% uranyl acetate (EMS). Uranyl acetate was removed by washing in ddH_2_O for 5 × 3 min and subsequently dehydrated and embedded as indicated above. Embedded samples were then mounted on aluminium s.e.m. stubs (diameter 12 mm) and coated with ∼8 nm of platinum (Quorum Q150T ES). Focused ion beam–scanning electron microscope imaging was performed using a Zeiss Auriga Crossbeam system with Atlas3D software. The focused ion beam was set to remove 5-nm sections by propelling gallium ions at the surface. Imaging was done at 1.5 kV using an ESB (back-scattered electron) detector. Three-dimensional reconstruction and segmentation were generated from the images stacks using Fiji ImageJ (NIH, USA) and ilastik softwares.

### APOL1 import into outer mitochondrial membrane

Standard methods for protein import into isolated mitochondria were followed^39^. Various *APOL1* PCR products were cloned into the *Kpn*I and *Xho*I restriction sites of the pYES2 vector (Invitrogen) with the decahistidine coding sequence between the *Xho*I and *Sph*I restriction sites. Radiolabelled APOL1 was synthesized in reticulocyte lysate (TNT T7 Coupled Reticulocyte Lysate System, Promega, L4610) in the presence of ^35^S-methionine and incubated with or without isolated mitochondria. The samples contained BSA buffer (3% (w/v) BSA, 80 mM KCl, 10 mM MOPS-KOH, pH 7.2), 2 μl reticulocyte lysate, 2 mM NADH, 1 mM ATP, 20 mM potassium phosphate and 30 μg (yeast) or 40 μg (rat liver) mitochondrial protein in a total volume of 100 μl. The import reactions were carried out for 10 min at 25 °C. The samples were subsequently cooled on ice and proteinase K was added at different concentrations. Following incubation for 10 min at 0 °C, the protease was inactivated by 2 mM phenylmethylsulfonyl fluoride. Re-isolated mitochondria were analysed by SDS-PAGE and proteins were detected by autoradiography.

### Measurement of endosomal pH

We followed exactly the method previously described^8^. The accumulation of the weak base [^14^C] methylamine was employed to investigate the intracellular pH and the pH of acidic organelles. Cells (2–3 × 10^7^ ml^−1^) were incubated at 37 °C in HMI-9 medium in the presence of [^14^C] methylamine (0.1 μCi ml^−1^; 1.8 μM) for 40 min. The cells were separated from the medium by rapid centrifugation through a 200-μl oil layer (2:1, v/v, mixture of di-*N*-butylphthalate and di-*iso*-octylphthalate). A parallel experiment was performed to determine the intracellular volume and the amount of trapped extracellular probe present in the cell pellet by incubation with ^3^H_2_O (1 μCi ml^−1^) and [^14^C] carboxyinulin (0.1 μCi ml^−1^). The accumulation ratio was determined by dividing the intracellular concentration of the probe by the extracellular concentration. The cytoplasmic pH was assessed by measuring methylamine accumulation in the presence of chloroquine (0.3 mM) or bafilomycin (1.8 μM).

### Analysis of the fatty acid composition

Total lipid was extracted by chloroform:methanol, 1:2 (v/v) and chloroform:methanol, 2:1 (v/v). Pooled organic phase was subjected to biphasic separation by adding water. Organic phase was dried under N_2_ gas flux. Total lipid was further separated to neutral lipid and phospholipid by silica gel column. Neutral lipid was eluted by chloroform:acetone 4:1 (v/v) and phospholipid was eluted by methanol. Each lipid fraction was dried under N_2_ gas flux. Each lipid fraction was methanolized by 0.5 M methanoic HCl at 100 °C for 1 h. Fatty acid methyl ester was extracted by hexane. The hexane phase was analysed by gas–liquid chromatography (PerkinElmer) on a BPX70 (SGE) column. Fatty acid methyl esters were identified by comparison of their retention times with those of standards and quantified by the surface peak method using C15:0 fatty acid for calibration.

### Lipid chase experiments

Growing cells (10^6^ ml^−1^) were washed and resuspended in serum-free HMI-9 supplemented with 2 mg ml^−1^ lipid-free BSA for 30 min. The suspension was supplemented (v/v) with a final concentration of either 2 μM BODIPY FL C5-Ceramide (Life Technologies) or 0.5 μM TopFluor Cholesterol (Avanti) together with 6 mg ml^−1^ lipid-free BSA for 30 min at 37 °C. Parasites were then pelleted by centrifugation and resuspended in HMI-9+serum medium at 37 °C, and aliquots were taken at different time points for FACS analysis.

### Phylogenetic tree

The sequences were aligned using MUSCLE, the alignment was curated using Gblocks and MrBayes was used for the phylogenetic analysis.

## Additional information

**How to cite this article:** Vanwalleghem, G. *et al.* Coupling of lysosomal and mitochondrial membrane permeabilization in trypanolysis by APOL1. *Nat. Commun.* 6:8078 doi: 10.1038/ncomms9078 (2015).

## Supplementary Material

Supplementary Information Supplementary Figures 1-11, Supplementary Tables 1-2 and Supplementary References

Supplementary Movie 1 FIB-SEM tomography of wild-type (WT) Trypanosoma brucei (white: mitochondrion; blue: kinetoplast; green: nucleus).

Supplementary Movie 2 FIB-SEM tomography of APOL1-treated wild-type (WT) *Trypanosoma brucei* (1 h-incubation with 10 μg ml-1 rAPOL1) (white: mitochondrion; blue: kinetoplast; green: nucleus).

Supplementary Movie 3 FIB-SEM tomography of a doxycycline-induced TbMFNL RNAi cell line of *Trypanosoma brucei* (2 days-incubation with 1 μg ml-1 doxycycline) (white: mitochondrion; blue: kinetoplast; green: nucleus).
