# Supplementary material for: Coupling of lysosomal and mitochondrial membrane permeabilization in trypanolysis by APOL1
Source: Nat Commun. 2015 Aug 26;6:8078. doi: 10.1038/ncomms9078 (PMC4560804; doi:10.1038/ncomms9078)
Supplement: Supplementary Information — Supplementary Figures 1-11, Supplementary Tables 1-2 and Supplementary References [file ncomms9078-s1.pdf]

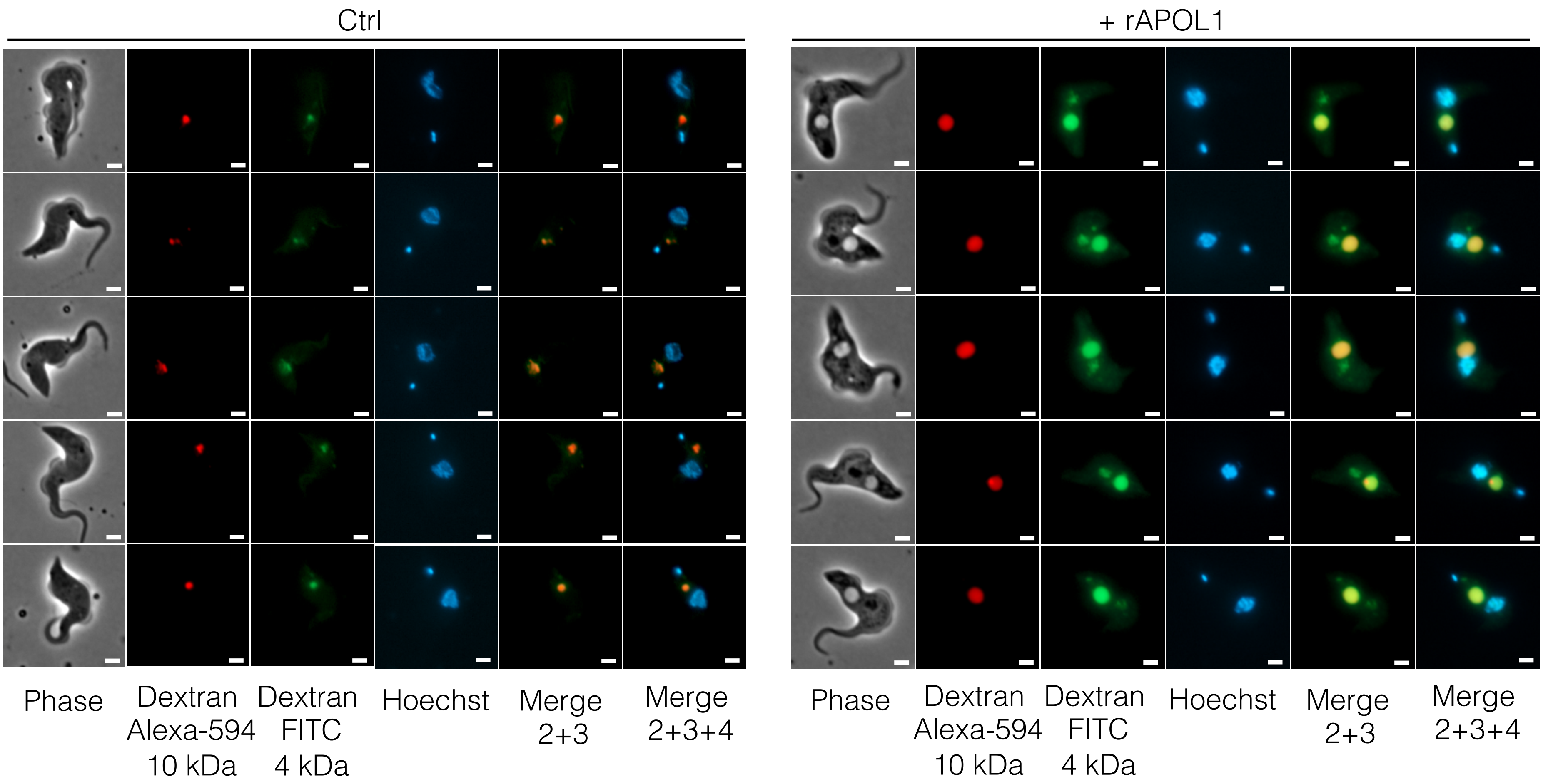

Supplementary Fig. 1. APOL1-induced LMP. Intracellular localization of 10 kDa- and 4 kDa- dextran beads in untreated (Ctrl) and 10 µg ml^{-1} rAPOL1-treated *T. brucei* (90 min incubation). The same exposure time was applied to all panels.

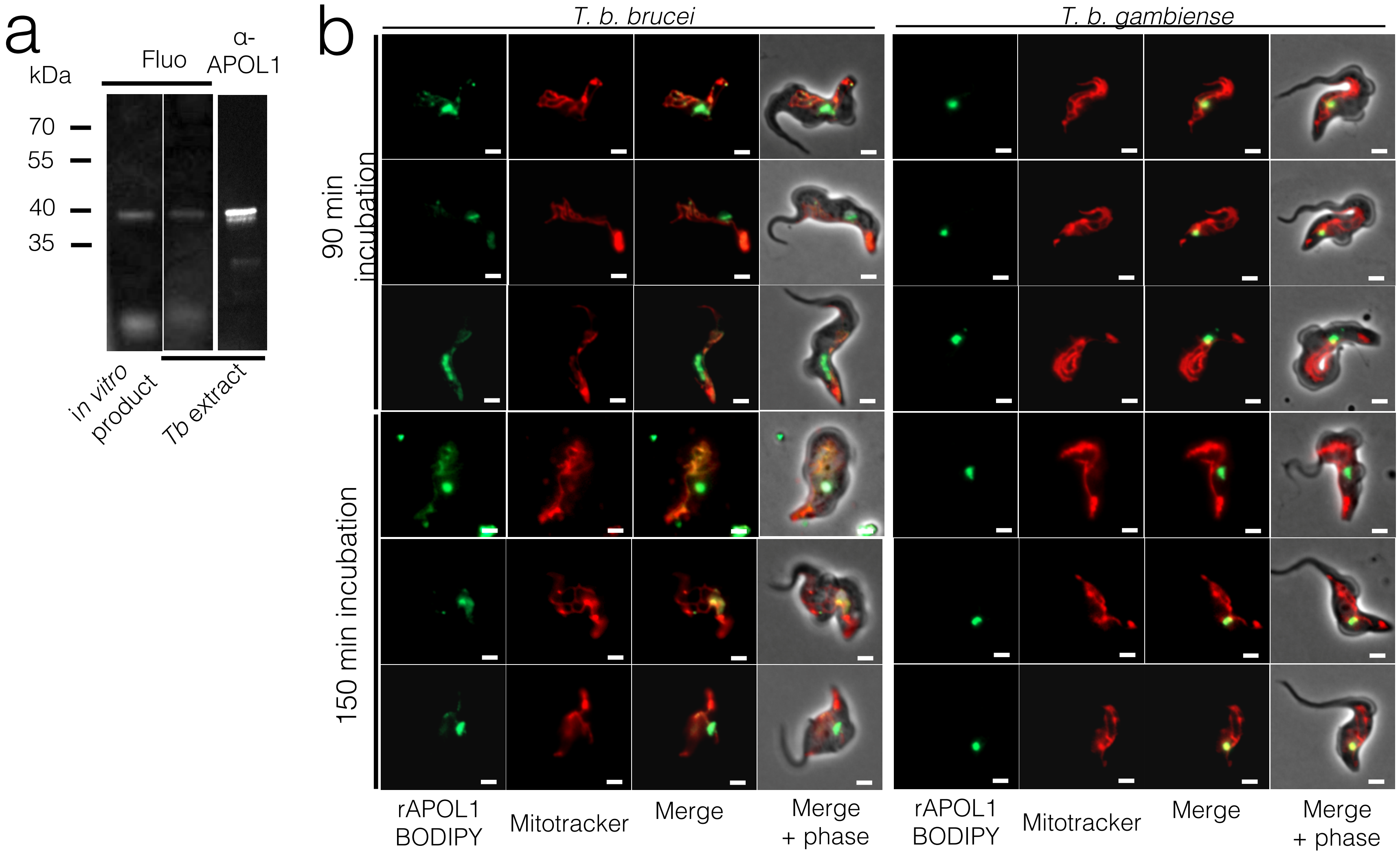

Supplementary Fig. 2. Intracellular localization of the rAPOL1-BODIPY conjugate in *T. b. brucei* and *T. b. gambiense*. (**a**) SDS-PAGE analysis of the BODIPY-conjugated rAPOL1, after synthesis or after isolation from trypanosomes following incubation for 90 min in the presence of the cysteine protease inhibitor FMK-024. (**b**) Intracellular localization of the rAPOL1-BODIPY conjugate. The mitotracker reveals the mitochondrion independently of membrane depolarization (bars=1 μm).

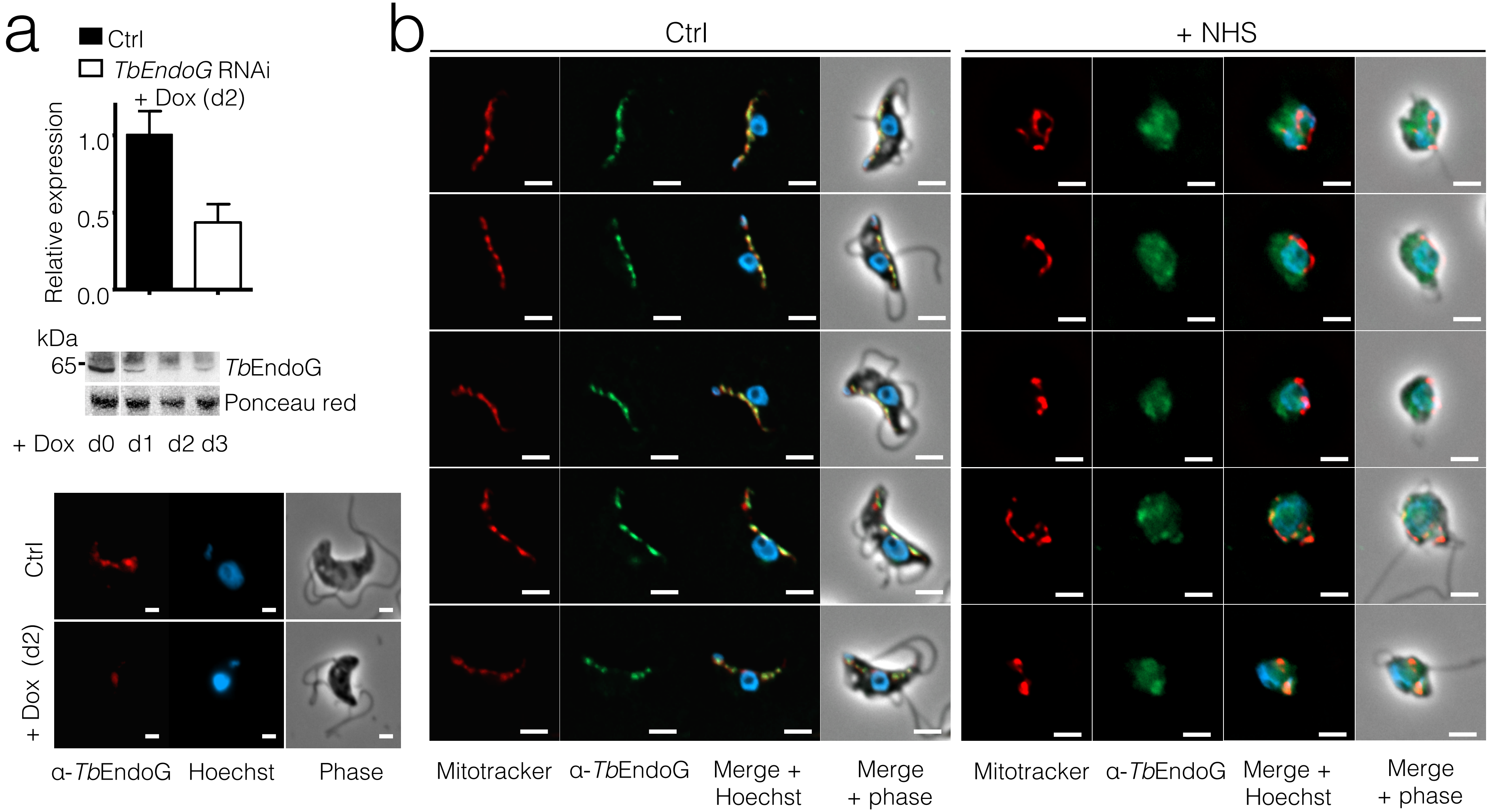

Supplementary Fig. 3. Expression and subcellular localization of *Tb*EndoG under different conditions. (**a**) *TbEndoG* down-regulation after *TbEndoG* RNAi induction by doxycycline (Dox), as measured by qRT-PCR (error bars represent s.e.m.; 3 replicates; n=3), Western blotting and immunofluorescence (in representative cells) (bars=2 µm). (**b**) Intracellular localization of *Tb*EndoG in *T. brucei,* untreated (Ctrl) or treated with 30% NHS (representative examples). The mitotracker identifies the mitochondrion, and the Hoechst dye labels the nucleus and kinetoplast DNA (large and small blue dot, respectively) (bars=2 µm).

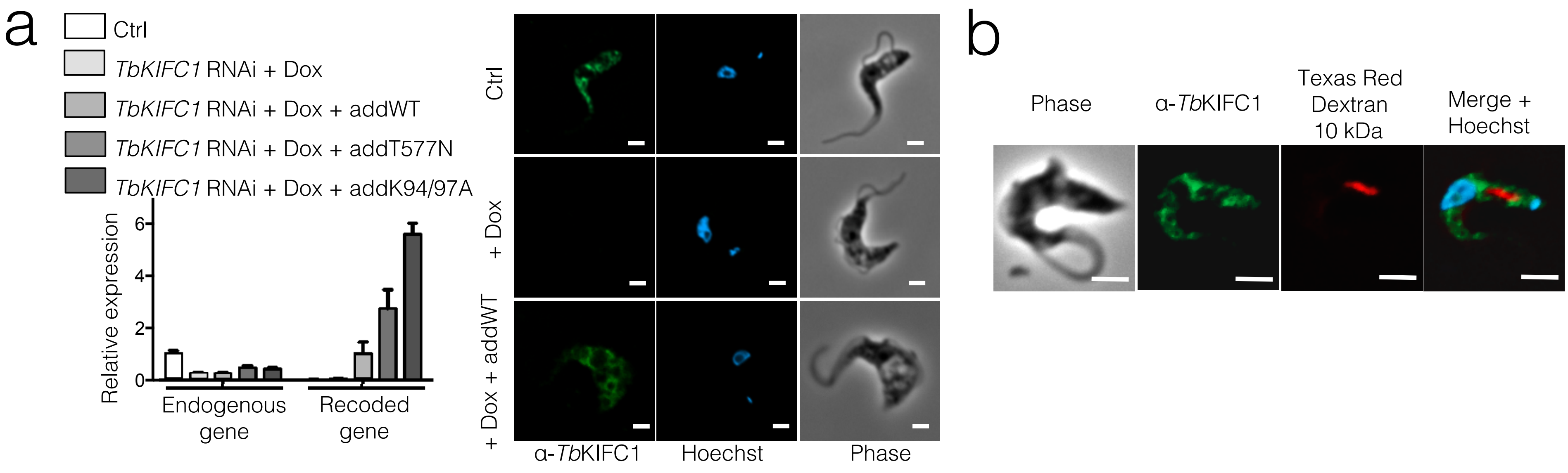

Supplementary Fig. 4. Expression of *Tb*KIFC1. (**a**) *TbKIFC1* down-regulation as measured in the RNAi cell line transfected or not with recoded gene constructs (addWT, wild-type gene; addT577N and addK94/97A, mutant genes), using qRT-PCR (error bars represent s.e.m.; 3 replicates; n=3) and immunofluorescence (in representative cells; bars=2 µm) after Dox induction (1 day). (**b**) Immunodetection of *Tb*KIFC1 using endocytosed 10 kDa dextran beads to visualize the endocytic compartment (representative example; bars=2 µm).

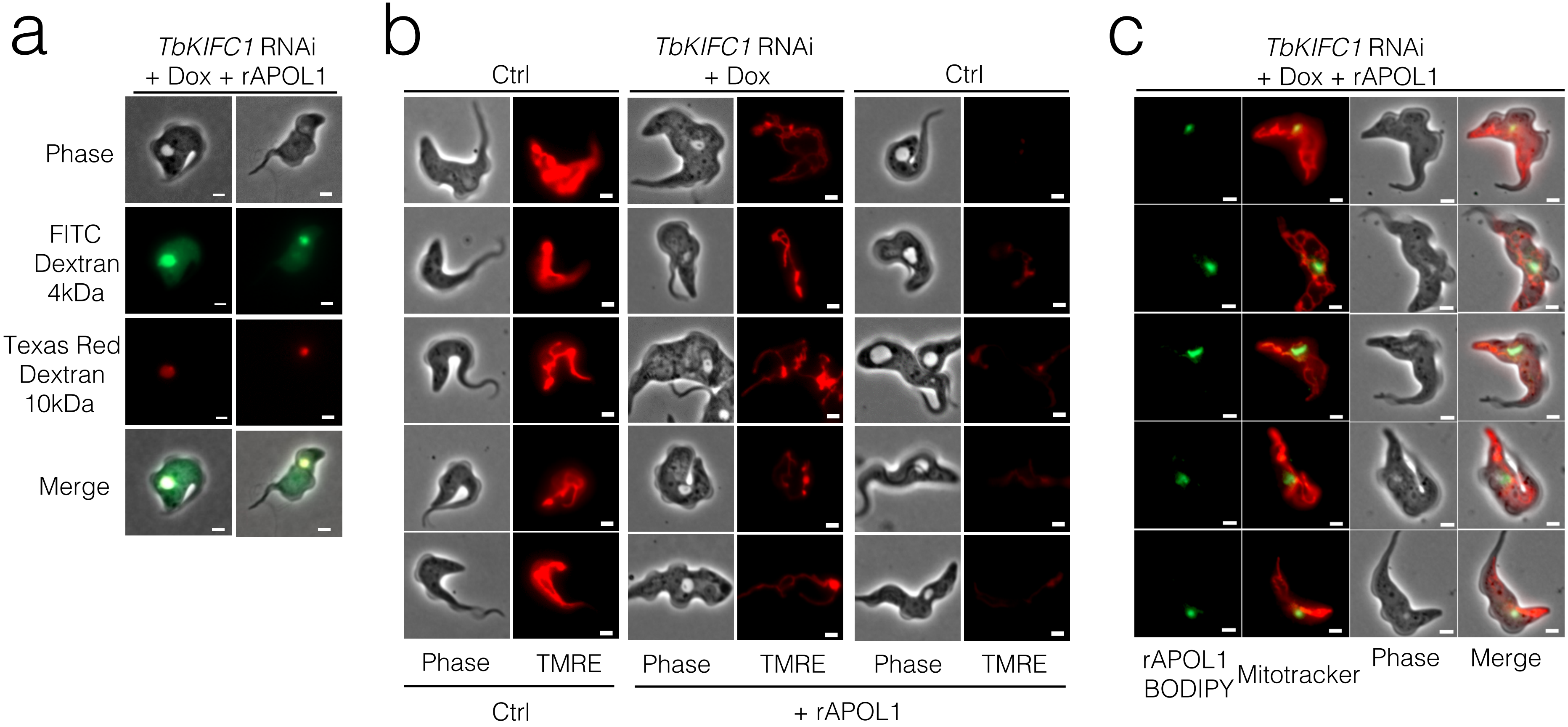

Supplementary Fig. 5. rAPOL1-triggered LMP and MMP in control (Ctrl) and Dox-induced *TbKIFC1* RNAi cells (1 day) (representative examples; bars=2 µm). (**a**) Intracellular localization of 4 and 10 kDa dextran beads in Dox-induced *TbKIFC1* RNAi cells treated for 1 h with 10 µg ml^{-1} rAPOL1. (**b**) TMRE staining of the mitochondrial membrane in control (Ctrl) and Dox-induced *TbKIFC1* RNAi cells treated or not for 1 h with 10 µg ml^{-1} rAPOL1. (**c**) Intracellular localization of the BODIPY conjugate of rAPOL1 incubated for 1 h with the *TbKIFC1* RNAi cell line after Dox induction.

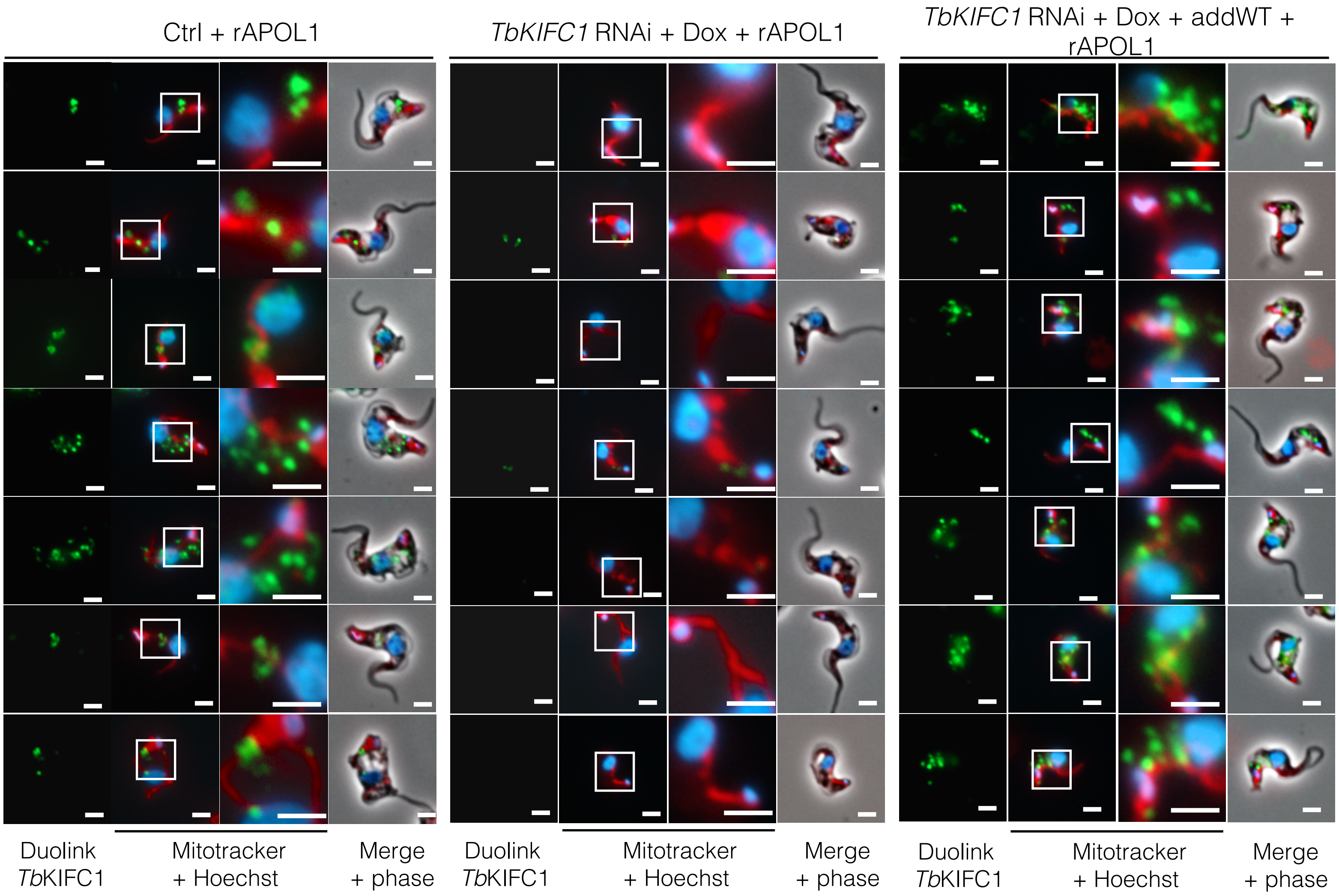

Supplementary Fig. 6. Duolink ligation proximity assay[18] between *Tb*KIFC1 and rAPOL1 in control and Dox-induced *TbKIFC1* RNAi cells (1 day) transfected or not with recoded addback gene construct, and treated or not for 1 h with 10 µg ml^{-1} rAPOL1 (representative examples; bars=2 µm). The mitotracker was used to localize the mitochondrion.

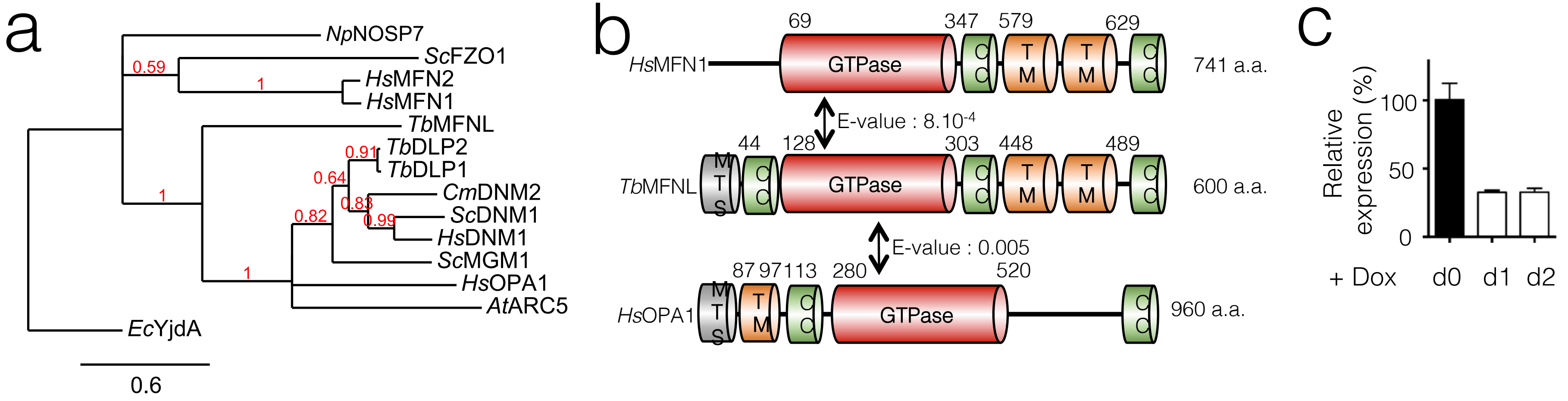

Supplementary Fig. 7. A trypanosomal mitofusin-like protein encoded by the Tb927.7.2410 gene (UniProt reference Q57XN3), termed *TbMFNL* in this work. (**a**) Phylogenic tree of mitofusin-related proteins (numbers on nodes indicate posterior probabilities; UniProt nomenclature: *Np*NOSP7= B2IZD3; *Sc*FZO1= P54861; *Hs*MFN1 = Q8IWA4; *Hs*MFN2 = O95140; *Tb*DLP1 = Q582R3; *Tb*DLP2 = Q582Q9; *Cm*DNM1 = M1VAR7; *Sc*DNM1 = Q6FJH5; *Hs*DNM1 = Q05193; *Sc*MGM1 = P32266; *Hs*OPA1 = O60313; *At*ARC5 = Q84N64; *Ec*YjdA = B7M8L9). (**b**) Sequence and structure comparison between *Tb*MFNL and human mitofusin 1 (*Hs*MFN1) and OPA 1 (*Hs*OPA1). E-values of BLAST are indicated between the GTPase domains. MTS=mitochondrial targeting sequence; CC=coiled-coil region; TM=transmembrane span. (**c**) *TbMFNL* down-regulation by Dox-induced RNAi as measured by qRT-PCR (error bars represent s.e.m.; 3 replicates; n=3).

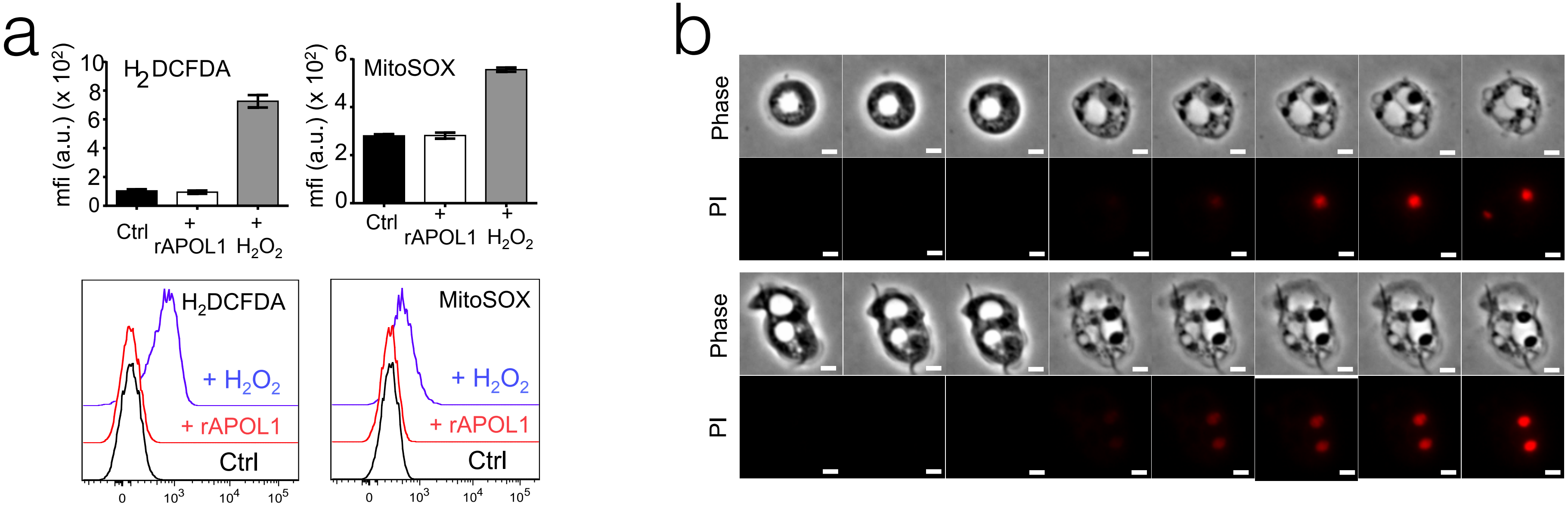

Supplementary Fig. 8. Components and pathways not playing a major role in APOL1-mediated LMP and MMP. (**a)** Involvement of reactive oxygen species in APOL1-induced trypanolysis. Measurement of oxidative stress in cells either treated with 10 µg ml^{-1} rAPOL1 for 1 h or with 10 µM H_2O_2 for 10 min, using flow cytometry after cellular staining with the general oxidative stress fluorescent indicator H_2DCFDA or the mitochondrial superoxide fluorescent indicator MitoSOX (mfi= mean fluorescent intensity of 10,000 cells; error bars represent s.e.m.; 3 replicates; n=3). (**b)** Detection of plasma membrane pores using propidium iodide (PI; 668 Da) during trypanolysis by rAPOL1 (bars=2 µm). Trypanosomes immobilized on agarose were incubated for 3 h with 10 µg ml^{-1} rAPOL1, then a time-laps sequence of 1 h was captured every 20 sec with 10 µg ml^{-1} PI. Frames were selected during the cell death event.

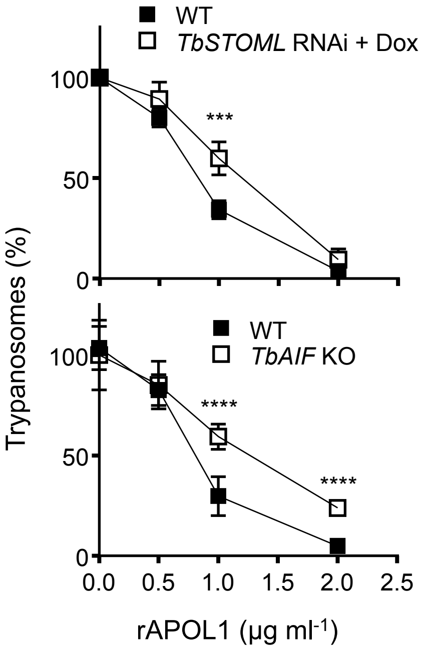

Supplementary Fig. 9. Components and pathways not playing a major role in APOL1-mediated LMP and MMP. Effect of *TbSTOML* RNAi induction for 2 days by Dox and effect of *TbAIF* KO on trypanolysis by rAPOL1 after overnight incubation (error bars represent s.e.m.; 3 replicates; n=10 and 12 for *Tb*STOML and *Tb*AIF respectively).

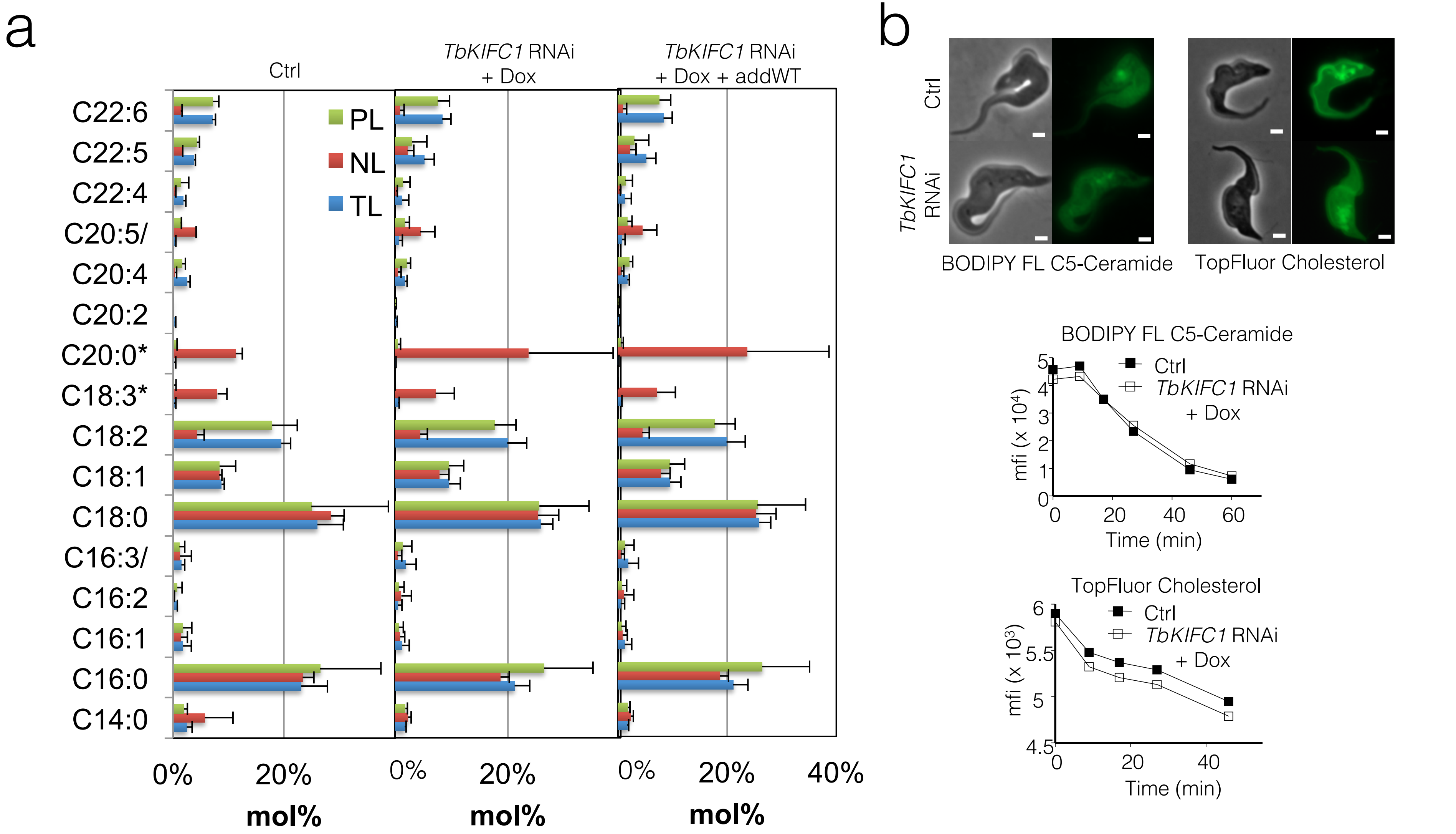

Supplementary Fig. 10. Lack of *Tb*KIFC1 involvement in the intracellular composition or trafficking of lipids. (**a**) Fatty acid composition of control (Ctrl) and Dox-induced *Tb*KIFC1 RNAi cells (1 day) transfected or not with recoded addback gene construct (TL, NL and PL designate total lipid, neutral lipid and polar lipid fractions respectively). (**b**) Sphingolipid and cholesterol turnover in control (Ctrl) and Dox-induced *TbKIFC1* RNAi cells (1 day), as measured by chase experiments after trypanosome incubation with 2 µM BODIPY® FL C5-Ceramide or 0.5 µM Top Fluor cholesterol. Panels at the top show the fluorescence of representative cells (bar=2 µm), and panels at the bottom show representative mfi kinetics of 10,000 cells/time point (n=3).

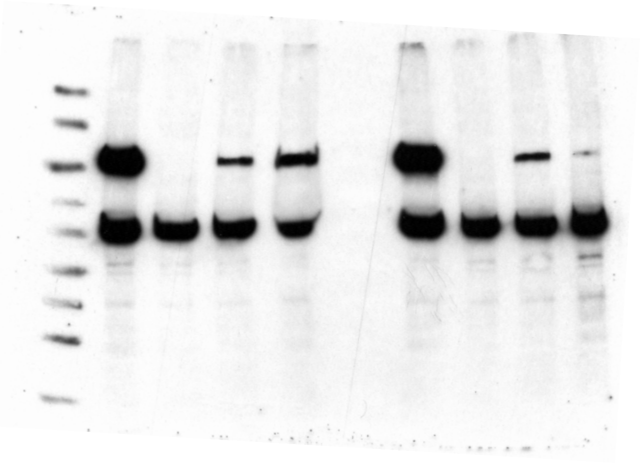

Fig. 5c

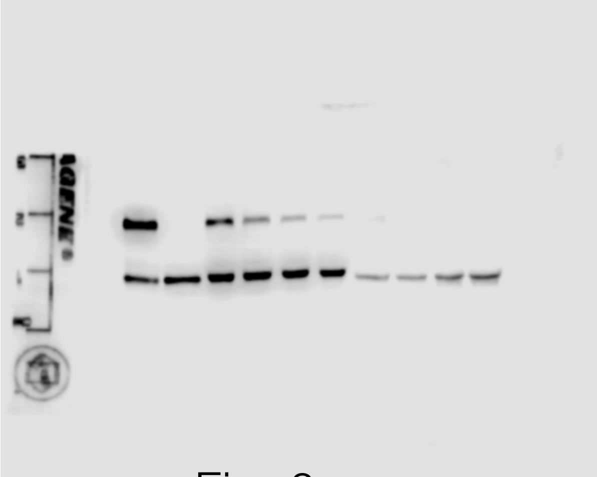

Fig. 6e

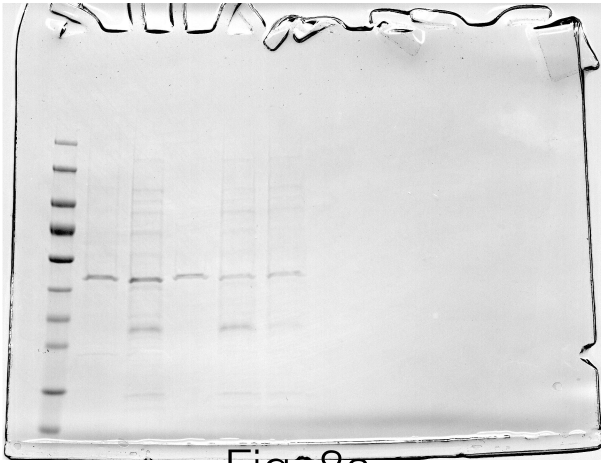

Fig. 8a

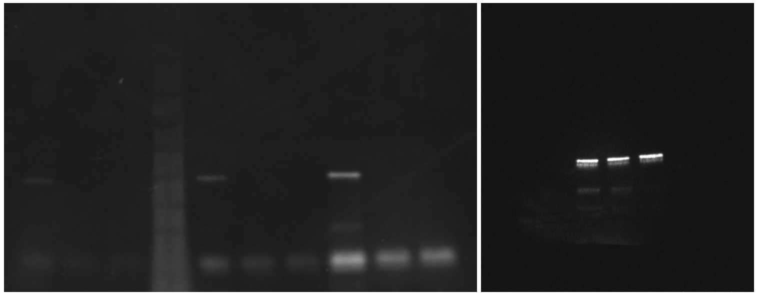

Suppl. Fig. 2a

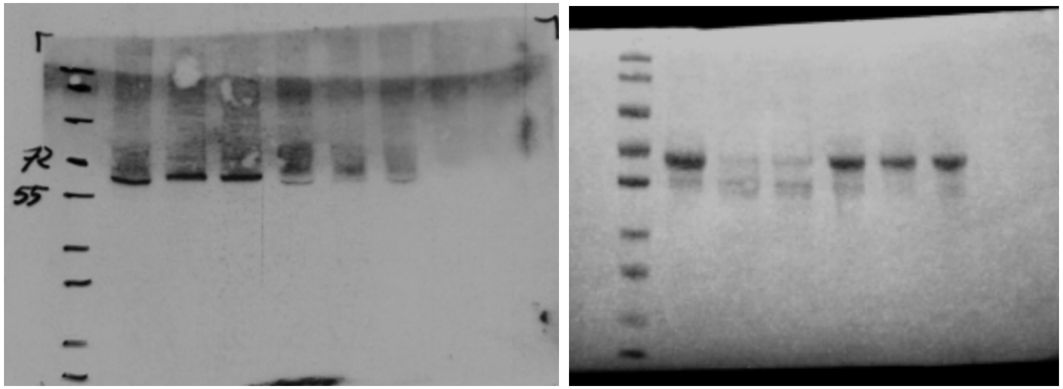

Suppl. Fig. 3a

Supplementary Fig. 11. Full images of the blots whose sections are shown in the Figures.

Cell	No addition	Chloroquine	Bafilomycin
Ctrl	18.7 ± 3.1	3.9 ± 1.1	3.8 ± 0.5
+ Dox	17.0 ± 0.7	4.9 ± 0.4	3.8 ± 0.1
+ Dox + addWT	17.3 ± 1.5	4.4 ±0.1	3.6 ± 0.2

Supplementary Table 1. Involvement of *Tb*KIFC1 in endosomal pH. Effect of Dox-induced *TbKIFC1* RNAi (1 day) on the accumulation of [^{14}C] methylamine, determined as previously described[8] (error bars represent s.e.m.; 4 replicates; n=1 except in control (Ctrl) cells where n=5). The accumulation ratio measured in the presence of chloroquine (0.3 mM) or bafilomycin (0.32 µM) was used as an indication of probe accumulation in response to the pH gradient across the plasma membrane, since under these conditions the pH gradient across acidic intracellular organelles (endosomal/lysosomal compartments) was abolished.

name	accession number	E-value	% Ctrl o/n resistance at 1 µg ml^{-1} rAPOL1	p-value of o/n resistance at 1 µg ml^{-1} rAPOL1	% mRNA level (Dox d2)	in mito-chondrial proteome (ref. 48)
*Tb*DLP	*Tb*927.3.4720 & *Tb*927.3.4760	2.10^{-153} to *Sc*DNM1p	90.2	0.9875	25.5	yes
*Tb*PHB 1-2	*Tb*927.8.4810 & *Tb*927.10.4310	2.10^{-78} & 3.10^{-73} to *Sc*PHB2p	96.8	> 0.9999	17.4 & 57.8	yes
*Tb*STOML	*Tb*927.5.520	3.10^{-78} to *Hs*STOML2	165.0	< 0.0001	41.2	yes
*Tb*SEY1	*Tb*927.10.14510	9.10^{-62} to *Sc*SEY1p	158.0	0.1134	17.3	yes
*Tb*MIRO	*Tb*927.8.560	6.10^{-9} to *Sc*GEM1p	115.7	0.9393	52.1	yes
*Tb*LDK	*Tb*11.01.0670	6.10^{-38} to *Hs*SIK3	105.2	0.9922	39.6	no
*Tb*AIF	*Tb*927.7.4310	2.10^{-5} to *Sc*AIF1p	227.5	< 0.0001	0 (KO)	yes

Supplementary Table 2. Effect on APOL1-induced trypanolysis, of downregulation of genes supposedly involved in intracellular lipid trafficking or apoptosis. Six top lines: lipid trafficking (*Tb*DLP, Dynamin related protein[41]; *Tb*PHB, Prohibitin[42]; *Tb*STOML, Stomatin-like protein[43]; *Tb*SEY1, ER Dynamin-like GTPase[44]; *Tb*MIRO, ER mitochondria encounter structure (ERMES) complex GTPase[45]; *Tb*LDK, Lipid droplets kinase[46]). Bottom line: apoptosis (Apoptosis Inducing Factor (AIF)[47].

Supplementary References

41. Chanez, A. L., Hehl, A. B., Engstler, M. & Schneider, A. Ablation of the single dynamin of T. brucei blocks mitochondrial fission and endocytosis and leads to a precise cytokinesis arrest. J. Cell Sci. 119, 2968–2974 (2006).

42. Ty´c, J. et al. Probing for primary functions of prohibitin in Trypanosoma brucei. Int. J. Parasitol. 40, 73–83 (2010).

43. Christie, D. A., Kirchhof, M. G., Vardhana, S., Dustin, M. L. & Madrenas, J. Mitochondrial and plasma membrane pools of stomatin-like protein 2 coalesce at the immunological synapse during T cell activation. PLoS One 7, e37144 (2012).

44. Voss, C., Lahiri, S., Young, B. P., Loewen, C. J. & Prinz, W. A. ER-shaping proteins facilitate lipid exchange between the ER and mitochondria in S. cerevisiae. J. Cell Sci. 125, 4791–4799 (2012).

45. Kornmann, B., Osman, C. & Walter, P. The conserved GTPase Gem1 regulates endoplasmic reticulum-mitochondria connections. Proc. Natl Acad. Sci. USA 108, 14151–14156 (2011).

46. Flaspohler, J. A., Jensen, B. C., Saveria, T., Kifer, C. T. & Parsons, M. A novel protein kinase localized to lipid droplets is required for droplet biogenesis in trypanosomes. Eukaryot. Cell 9, 1702–1710 (2010).

47. Wissing, S. et al. An AIF orthologue regulates apoptosis in yeast. J. Cell Biol. 166, 969–974 (2004).

48. Niemann, M. et al. Mitochondrial outer membrane proteome of Trypanosoma brucei reveals novel factors required to maintain mitochondrial morphology. Mol. Cell Proteomics 12, 515–528 (2013).
